# Monocyte migration assay using a vascular-on-a-chip model and its utilization for the evaluation of a heated tobacco product

**DOI:** 10.3389/ftox.2025.1658093

**Published:** 2025-12-04

**Authors:** Ayaka Hayashida, Atsuko Nozawa, Shigeaki Ito

**Affiliations:** Scientific Product Assessment Center, Japan Tobacco, Inc., Yokohama, Kanagawa, Japan

**Keywords:** organ-on-a-chip, atherosclerosis, cardiovascular disease, monocyte migration, heated tobacco products, macrophages, new approach methodologies

## Abstract

The use of Heated Tobacco Products (HTPs) is expected to have a reduced-risk potential for cardiovascular disease, including atherosclerosis, compared with combustible cigarettes (CCs) Because of the complex relationship between atherosclerosis and lifestyle factors, such as diet, physical activity, and smoking, focusing on the pathogenesis of atherosclerosis will help deepen our understanding of the reduced risk potential of HTPs. Organ-on-a-chip platforms are widely used to mimic human pathophysiology when studying such pathologic manifestations. In this study, a Vascular-on-a-Chip (VoC) model was used to mimic the characteristic physiology of the human vasculature and to establish an assessment model to measure three endpoints: endothelial barrier impairment, monocyte adhesion, and monocyte migration through vascular endothelial cells (VECs) which are the important initial key events in atherosclerosis. Macrophages were exposed to test cigarette smoke (CS) and HTP aerosol extracts, and conditioned medium was collected. VECs cultured on VoC were exposed to these conditioned media to mimic the effects on the vascular system caused by inflammatory responses elicited by inhaled substances. The HTP aerosol-exposed group had significantly reduced endothelial barrier impairment, monocyte adhesion, and monocyte migration compared with the CS-exposed group, and there was no significant difference with the solvent control. In summary, our model provided valuable insights into the reduced risk potential of an HTP compared with a CC by evaluating a series of endpoints in the early stage of atherosclerosis.

## 1 Introduction

Atherosclerotic cardiovascular disease (CVD) is the dominant cause of morbidity and mortality worldwide (GBD, 2017 Cau ses of Death Collaborators, 2018). Atherosclerosis is initiated through chronic inflammation and oxidative stress, eventually leading to morbidity symptoms such as myocardial infarction and stroke related to plaque formation, thrombosis, and arterial narrowing (Marchio et al., 2019; Higashi, , 2022). Recent comprehensive reviews have revealed that various molecular events caused by long-lasting inflammation in vascular regions play crucial roles in the progression of atherosclerosis (Kong et al., 2022; Attiq et al., 2024). In the initial stage of atherosclerosis, proinflammatory cytokines promote endothelial dysfunction, including increased endothelial permeability and the expression of adhesion molecules (*e.g.*, E-selectin, intercellular adhesion molecule-1, and vascular cell adhesion molecule-1) (Mirjana, 2022). Monocytes in the blood captured by these adhesion molecules roll on the surface of vascular endothelial cells (VECs) and migrate through the damaged VEC barrier, attracted by chemotactic factors such as monocyte chemotactic protein-1 (MCP-1, also known as CCL2). These migrated monocytes then differentiate into macrophages (MΦs), which take up oxidized low-density lipoprotein to form foam cells (Moroni et al., 2019). Chronic inflammation promotes the secretion of matrix metalloproteinases from cells in the vascular wall (*e.g.*, vascular smooth muscle cells, immune cells, VECs) (Newby, 2005), leading to thinning of the plaque fibrous cap and subsequently increasing the risk of plaque rupture (Popa-Fotea et al., 2023). Anti-inflammatory treatments that target interleukin (IL)-1β, a representative proinflammatory cytokine, were reported to significantly reduce the rate of recurrent cardiovascular events (Ridker et al., 2017). An *in vivo* study revealed that a deficiency in another major inflammatory cytokine, tumor necrosis factor (TNF)-α, reduced the onset of atherosclerosis (Branen et al., 2004). These data suggest that excessive inflammation accelerates atherosclerosis onset and progression; thus, the suppression of inflammation might contribute to reducing the risk of atherosclerosis.

Smoking is considered a risk factor for atherosclerosis. Harmful and potentially harmful constituents (HPHCs) present in tobacco smoke, which are thought to be potential drivers of inflammatory responses (Centers for Disease Control and Prevention, 2010), have been suggested to exert deleterious effects on cardiovascular health. In recent years, Heated Tobacco Products (HTPs) have emerged, and several studies have reported that HTP aerosol contained fewer substances and lower levels of HPHCs in their emissions compared with cigarette smoke (CS) because HTPs generate aerosols by heating, rather than by burning tobacco (Schaller et al., 2016; Forster et al., 2018; Takahashi et al., 2025; Hashizume et al., 2023). These reports also showed that exposure to HTP aerosol had a lower biological impact on various outcomes *in vitro* and *in vivo* tests compared with CS exposure (Schaller et al., 2016; Hashizume et al., 2023; Wang et al., 2021; Chapman et al., 2023; Kogel et al., 2015; Wong et al., 2016). As atherosclerosis-relevant endpoints, Phillips et al. reported that ApoE^−/−^ mice exposed to an HTP aerosol developed fewer atherosclerotic plaques compared with mice exposed to CS (Phillips et al., 2019). However, further investigation is required to clarify the potential impact of HTPs on an individual’s atherosclerosis risk and overall public health.

Currently, there is an increasing interest in mechanism-based, high-throughput testing systems using New Approach Methodologies (NAMs). The use of NAMs could provide valuable insights to help elucidate and validate the reduced risk potential of HTPs for tobacco-related disease (Thorne et al., 2024). Organ-on-a-Chip (OoC) technologies, an innovative type of NAM, allow evaluation of the organ-specific effects of test substances on humans under more physiologically relevant environments compared with conventional *in vitro* models. Vascular-on-a-chip (VoC) is expected to recapitulate the physiological conditions of the actual vasculature, where shear stress and turbulence can be reproduced by mimicking the blood flow in a microfluidic system. Unlike conventional static *in vitro* models, VoC enables *in vitro* risk assessment to be performed under dynamic blood flow conditions, which influence atherosclerosis development. Previously, we reported the use of VoC to assess monocyte adhesion, an important initial key event in atherosclerosis (Ohashi et al., 2023). The VoC model used in that study maintained VECs under constant and low shear stress (<1.41 dyne/cm^2^) during culture. This shear stress was similar to that observed in the atherosclerosis-prone regions of human arteries (<10 dyne/cm^2^) (Bacigalupi et al., 2024; Corban et al., 2014). Therefore, the VoC model can be used to evaluate biological events under conditions close to the human vasculature. Monocyte adhesion is an initial event in the development of atherosclerosis, and using the VoC, we showed that HTP aerosol induced less monocyte adhesion than CS, even under atherosclerotic prone conditions, possibly because of its lower inflammatory potential. The Organization for Economic Co-operation and Development proposes an adverse outcome pathway (AOP) framework to assess toxicological effects with a sequential chain of causally linked events that lead to an adverse health effect (OECD, 2017). The AOP approach is expected to be useful for toxicological assessments as well as evaluating disease risk based on the mechanisms of onset and progression. Following this approach, it is necessary to assess early events and the subsequent events triggered by HTP aerosol exposure for a better understanding of the reduced risk potential of HTPs for atherosclerosis.

During the pathogenesis of atherosclerosis, monocyte migration follows monocyte adhesion, and monocytes that have adhered to the surface of VECs migrate into the intima region by following a chemokine gradient (Ley et al., 2011). Gosling et al. reported the reduced migration of immune cells and a subsequent significant decrease around atherosclerotic lesions in MCP-1-deficient mice compared with control mice (Gosling et al., 1999). Therefore, evaluating the impact of CS and HTP aerosol exposure on monocyte migration will provide useful insights to help interpret their biological impact on atherosclerosis following the AOP-based toxicological assessment. In this study, we advanced our assessment model using VoC by evaluating monocyte adhesion, as well as the impaired barrier integrity of VECs and monocyte migration, sequentially in a chip under microfluidic conditions. We hypothesized that this assessment model using VoC would reproduce cytokine/chemokine-inducible monocyte migration into the intima region. In addition, the assessment model could reflect the inflammatory conditions present during atherosclerosis development. It is therefore potentially useful for the comparative CVD risk assessment of CS and HTP aerosol. We expect that our model will provide mechanistic insights into the reduced-risk potential of HTPs in relation to CVD.

## 2 Materials and methods

### 2.1 Preparation of CC smoke and DT3.0a aerosol extracts

CC and Direct Heating Tobacco System Platform 3 Generation 0 version a (DT3.0a) were used for the following assessments. The smoke or aerosol extracts were prepared as described previously (Ohashi et al., 2023). For CC, Kentucky reference 1R6F cigarettes were purchased from the University of Kentucky, Kentucky Tobacco Research and Development Center (Lexington, KY, United States). They were promptly stored at 4 °C upon arrival, stored at 4 °C until used, and used within 3 months to maintain their quality, in accordance with the manufacturer’s instructions (Center for Tobacco Reference Products, 2024). The DT3.0a device and representative regular flavor sticks are commercially available in Japan. The 1R6F cigarettes and tobacco sticks for DT3.0a were stored for at least 48 h at 22 °C ± 1 °C with 60% ± 3% relative humidity in accordance with the International Organization for Standardization (ISO) 3,402 standard (ISO, 2023). The battery of the DT3.0a heating device was fully charged before aerosol generation. 1R6F CS was generated with an RM20H smoking machine (Borgwaldt, Hamburg, Germany) in accordance with ISO 20778 (55 mL puff volume, 30-second puff interval, 2-second puff duration, bell-shaped puff profile, and 100% blocked ventilation holes) (ISO, 2018). DT3.0a aerosol was generated with an LM5E analytical vaping machine (Borgwaldt) in accordance with a modified ISO20778 (55 mL puff volume, 30-second puff interval, 2-second puff duration, bell-shaped puff profile, unblocked ventilation holes). The ventilation holes were unblocked because the position of the ventilation holes inside the device precluded the possibility of blocking ventilation holes under the intended conditions of use. Total particulate matter (TPM) from 1R6F CS and aerosol collected mass (ACM) from DT3.0a were collected using a 44-mm Cambridge filter pad separately. After quantifying the weight of the particulate phase collected on the filter pad, dimethyl sulfoxide (DMSO; FUJIFILM Wako Pure Chemical Corporation, Osaka, Japan) was added to extract the sample, yielding target concentrations of 40 mg/mL and 200 mg/mL for TPM and ACM, respectively. Summaries of the data measured after TPM or ACM collection are shown in Supplementary Table S1. The extracts of TPM and ACM were stored at −80 °C until used, and these extracts were used for testing within 1 year (Crooks et al., 2013).

### 2.2 Cell culture

Primary human coronary artery endothelial cells (HCAECs) were purchased from Promocell GmbH (Heidelberg, Germany) and cultured in a collagen-coated flask with Endothelial Cell Growth Medium MV2 (Promocell) containing 1% penicillin-streptomycin (FUJIFILM Wako Pure Chemical Corporation). HCAECs of passages 4–6 were used in experiments. The human acute monocytic leukemia cell line (THP-1 cells) was purchased from American Type Culture Collection (Manassas, VA, United States) and cultured in a non-coated flask with Roswell Park Memorial Institute (RPMI) 1,640 medium (Thermo Fisher Scientific, Waltham, MA, United States) containing 10% deactivated fetal bovine serum (FBS, Thermo Fisher Scientific, LOT 2534337) and 1% penicillin-streptomycin. THP-1 cells of passages 7–13 were used for tests.

### 2.3 OrganoPlate^®^ culture

OrganoPlate^®^ 3-lane 40 (MIMETAS B.V., Leiden, the Netherlands) was used for the VoC culture. A schematic overview of the OrganoPlate^®^ is shown in Supplementary Figure S1. Before cell seeding, 50 µL of cold Hank’s balanced salt solution (HBSS, Thermo Fisher Scientific) was dispensed into the observation window to obtain optical clarity. Eight volumes of 5 mg/mL Cultrex 3D collagen I rat tail (R&D Systems, Minneapolis, MN, United States) were mixed with one volume of 1 M 4-(2-hydroxyethyl)-1-piperazineethanesulfonic acid (HEPES, Thermo Fisher Scientific) and 37 g/L NaCO_3_ pH 9.5 aqueous solution on ice. This 4 mg/mL collagen I solution was mixed with Geltrex (Thermo Fisher Scientific) at a ratio of 4:1 on ice. If bubbles appeared in the collagen solution, the tube was centrifuged for about 10 s to remove them. The collagen solution was used within 15 min after mixing. Then, 2 µL of collagen was dispensed to the gel inlet, and the plate was incubated for 15 min at 37 °C with 5% CO_2_ to complete gelation. Concurrently, HCAECs were washed with 10 mL of warmed phosphate buffer solution (PBS, pH 7.4; Thermo Fisher Scientific), and then detached from the culture flask using 0.25% Trypsin-EDTA (Thermo Fisher Scientific). After 5 min of incubation at 37 °C with 5% CO_2_, HCAECs were resuspended in MV2 medium. After counting the density of cells, a suspension of HCAECs containing 2 × 10^6^ cells was aliquoted to another tube and centrifuged at 200 *g* for 5 min. After the supernatant was discarded, a cell pellet of 2 × 10^6^ cells was resuspended in 200 μL of OrganoMedium HUVEC-ABM (MIMETAS B.V.), and 2 μL of this cell suspension was applied to the medium inlet. After cell seeding, 50 μL of OrganoMedium was added to the medium inlet. The plate was incubated at an angle using the plate stand provided by MIMETAS for 3 h to allow the cells to attach to the collagen. Subsequently, 50 μL of OrganoMedium containing 200 μM GM6001 (Abcam, Cambridge, United Kingdom) was dispensed to the medium outlet to inhibit matrix degradation, and 50 μL of HBSS was dispensed in the bottom inlet and outlet, respectively. OrganoMedium was used as the culture medium for VoC to recreate the barrier function of HCAECs (Supplementary Figure S2). The plate was placed on an OrganoFlow^®^ (MIMETAS B.V.) set at a 7° angle and inverted every 8 min to reproduce bidirectional flow in the perfusion channel of the plate in an incubator. The plate was cultured for 4 days until the formation of the tubule structures of HCAECs was observed. Medium was replaced on day 3.

### 2.4 Macrophage differentiation

M1MΦs were differentiated from THP-1 cells by two stimulation steps. Initially, THP-1 cells were stimulated using differentiation medium 1: 100 nM phorbol 12-myristate 13-acetate (Sigma-Aldrich, St. Louis, MO, United States), 4 mM L-glutamine (Thermo Fisher Scientific), and 500 μM monothioglycerol (FUJIFILM Wako Pure Chemical Corporation) in Advanced RPMI 1640 (Thermo Fisher Scientific) containing 1% FBS. THP-1 cells were aliquoted at 1.5 × 10^7^ cells. After centrifugation of the cell suspension at 200 × g for 5 min, the supernatant was gently removed. THP-1 cells were resuspended in 75 mL of differentiation medium 1 to achieve a cell density of 2 × 10^5^ cells/mL and were seeded at 1 mL per well in a 24-well collagen-coated plate (Corning, Corning, NY, United States). After 72 h of incubation, differentiation medium 1 was replaced with 1 mL of fresh Advanced RPMI 1640 medium containing 1% FBS, and the plate was incubated for an additional 24 h to allow cells to rest. The second stimulation was performed in differentiation medium 2 containing 20 ng/mL interferon-γ (IFN-γ, FUJIFILM Wako Pure Chemical Corporation) and 10 pg/mL lipopolysaccharide (LPS, FUJIFILM Wako Pure Chemical Corporation) in Advanced RPMI 1640 medium containing 1% FBS. The Advanced RPMI 1640 medium in the plate was replaced with 1 mL of differentiation medium 2. After 24 h of stimulation, differentiation medium 2 was replaced with fresh Advanced RPMI 1640 medium containing 1% FBS, and the plate was incubated for an additional 16 h to allow cells to rest.

### 2.5 Preparation of conditioned medium from M1 macrophages

TPM and ACM extracts were diluted to 400 μg/mL with MV2 medium containing 1% DMSO. As control samples, MV2 containing 1% DMSO (solvent control) and MV2 containing 100 ng/mL LPS (positive control) were prepared. M1MΦs derived from THP-1 in a 24-well plate were exposed to 500 μL of each exposure sample for 1 h at 37 °C with 5% CO_2_. After treatment, all exposed samples received 1 mL of fresh OrganoMedium. After 3 h of incubation at 37 °C with 5% CO_2_, OrganoMedium containing secretions from M1MΦs exposed to each tobacco sample (hereafter referred to as conditioned medium) was collected and pooled. Debris and detached cells were removed from the conditioned medium by centrifugation at 200 *g* for 5 min, and then the supernatants were collected. Hereafter, these conditioned media are referred to as 1R6F TPM-, DT3.0a ACM-, 1% DMSO-, and LPS-conditioned medium, respectively, corresponding to the samples to which MΦs were exposed. The collected test samples were stored at −80 °C until used.

### 2.6 Sample exposure to VoC

Human recombinant IL-1β (FUJIFILM Wako Pure Chemical Corporation) or human recombinant TNF-α (FUJIFILM Wako Pure Chemical Corporation) were diluted with OrganoMedium to prepare a concentration range from 100 pg/mL to 10 ng/mL. To establish a monocyte migration assay under inflammatory conditions, HCAECs were exposed to 50 μL of the diluted solution, including each cytokine at the medium inlet and outlet. Conditioned media prepared from each tobacco sample were diluted 2-fold with OrganoMedium and exposed to HCAECs in the same way as proinflammatory cytokines. The HCAECs were incubated for 24 h at 37 °C with 5% CO_2_ on the OrganoFlow^®^ at a 7° angle with inversion every 8 min.

### 2.7 Trans-endothelial electrical resistance measurement

OrganoTEER^®^ (MIMETAS B.V.) was used to quantify the barrier integrity of HCAECs cultured in the OrganoPlate^®^. Before measurements, 50 μL of pre-warmed OrganoMedium was added to the gel inlet and outlet and then the plate was placed on a HIENAI Mat 01R (Cosmo Bio Company Limited, Tokyo, Japan) for 5 min to equilibrate the temperature of the media in the chips. The plate was applied to OrganoTEER^®^ equipment in accordance with the manufacturer’s instructions, and the trans-endothelial electrical resistance (TEER) value of each tubule in the plate was measured.

### 2.8 Monocyte adhesion and migration measurements

THP-1 monocytes were labeled with Calcein-AM solution (DOJINDO, Kumamoto, Japan) in RPMI 1640 medium for 15 min in an incubator (37 °C with 5% CO_2_). The fluorescent-labeled THP-1 monocytes were centrifuged at 200 *g* for 5 min to remove the supernatants and resuspended in OrganoMedium at 5 × 10^5^ cells/mL. MCP-1 (Sigma-Aldrich) was diluted to 1,000 ng/mL with OrganoMedium. After removing the medium in the top lane, 50 mL of fluorescent-labeled THP-1 cells was added to the medium inlet and outlet, and 1,000 ng/mL of MCP-1 solution in OrganoMedium was added to the bottom inlet and outlet. Two hours after the addition of THP-1 cells, monocyte adhesion was assessed by acquiring fluorescent images using Operetta CLS (Revvity, Waltham, MA, United States) to count THP-1 cells, which were present inside the tubes of HCAECs. Forty-eight hours after the addition of THP-1 cells, monocyte migration was assessed by acquiring fluorescent images to count the number of THP-1 cells present in the middle lane of OrganoPlate^®^ 3-lane 40. Image analyses were performed with Fiji (ImageJ version 1.54f) (Schindelin et al., 2012).

### 2.9 Cytokine measurement

Enzyme-linked immunosorbent assays (ELISA) were performed to detect the concentration of cytokines in conditioned medium. The concentrations of IL-1β and TNF-α in conditioned medium were measured using a Human IL-1 beta/IL-1F2 Quantikine ELISA kit (R&D Systems) and Human TNF-alpha Quantikine ELISA kit (R&D Systems), respectively, in accordance with the manufacturer’s instructions. The optical density at 450 nm in each well was detected by a Cytation5 reader (Agilent Technologies, Santa Clara, CA, United States).

### 2.10 Cytotoxicity assay

The cytotoxicity of MΦs exposed to test samples was detected by a Cell Counting Kit-8 (DOJINDO) according to the manufacturer’s instructions. Briefly, M1MΦs were exposed to 500 μL of prepared exposure samples for 1 h. After exposure, all exposure samples were replaced with 1 mL of fresh OrganoMedium. The absorbance at 450 nm of each well was measured by Cytation5 as the background, and then 100 μL of Cell Counting Kit-8 solution was added to each well and incubated for 3 h at 37 °C with 5% CO_2_. After gentle shaking of the plate, the absorbance at 450 nm of each well was measured. The background absorbance was subtracted from the absorbance after incubation to calculate cytotoxicity.

### 2.11 Immunofluorescence staining

Unless otherwise specified, all immunostaining procedures were performed at room temperature. HCAECs were fixed with 4% paraformaldehyde PBS (FUJIFILM Wako Pure Chemical Corporation) for 15 min. After washing with PBS, cells were permeabilized with 0.3% Triton X-100 (Sigma-Aldrich) in PBS for 10 min. After washing with washing solution (PBS containing 4% FBS), cells were treated with blocking solution (PBS containing 2% FBS, 2% bovine serum albumin (Sigma-Aldrich), and 0.1% polysorbate 20 (MP Biomedicals, Irvine, CA, United States)) for 45 min. Then, the blocking solution was replaced with primary antibody solution containing ZO-1 polyclonal antibody (Thermo Fisher Scientific, LOT ZE392355) at 2.5 μg/mL in blocking solution and incubated with cells overnight at 4 °C. After washing with washing solution, a secondary antibody solution containing goat anti-rabbit Alexa Fluor 594 (Abcam) at 2 μg/mL in blocking solution was added to each chip. After overnight treatment at 4 °C, each chip was washed with washing solution and dyed with Hoechst 33,342 solution (Thermo Fisher Scientific) diluted 2000-fold with blocking solution for 20 min. After washing with washing solution and replacement of the solution with PBS, immunofluorescence images were obtained by Operetta CLS (Revvity).

### 2.12 Statistical analysis

Because of the limited sample size and difficulty in confirming normality and homoscedasticity, non-parametric tests were performed. For multiple groups, pairwise comparisons following the Kruskal–Wallis test were performed using Dunn’s test with Bonferroni correction to adjust for multiple comparisons. For the comparison of two groups, the Mann–Whitney *U*-test was performed. The effect size was calculated by dividing the Z-score from the Mann–Whitney *U*-test by the square root of the number of biological replicates (Rosenthal, 1986; Field, 2013). Effect sizes were interpreted according to conventional thresholds: small (*r* = 0.1–0.3), medium (*r* = 0.3–0.5) and large (*r* > 0.5) (Cohen, 1988). All statistical analyses were performed using JMP software (version 18, SAS Institute, Cary, NC, United States). The number of biological replicates (n) within a plate and independent technical replicates (N) are indicated in the figure legends for each experiment. Statistical analyses were performed using independent biological replicates (n).

## 3 Results

### 3.1 Monocyte adhesion and migration under inflammatory conditions

**FIGURE 1:**
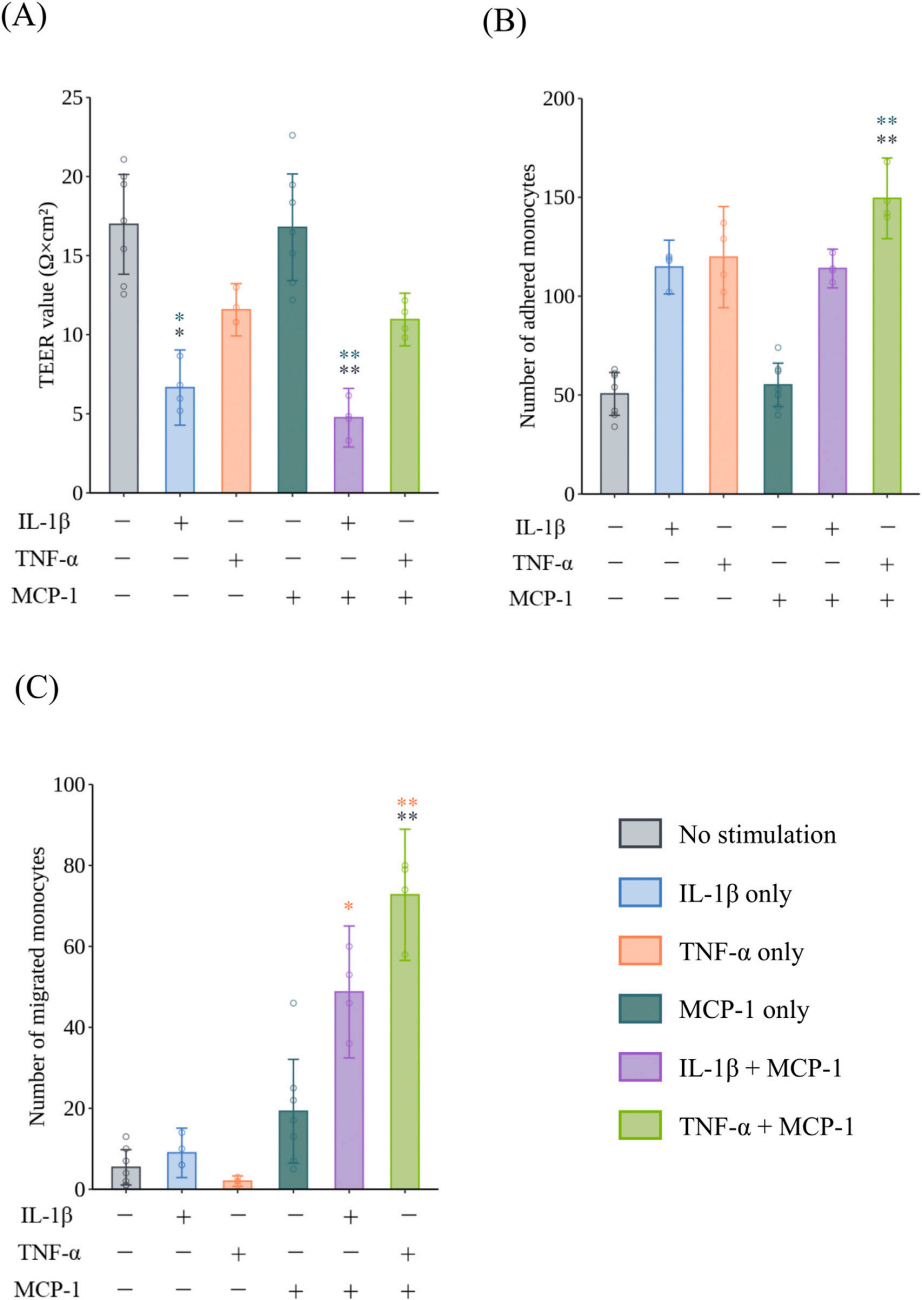
Barrier impairment, monocyte adhesion, and migration in VoC with or without proinflammatory cytokines and chemokines. The effect of 10 ng/mL IL-1β or 10 ng/mL TNF-α with or without MCP-1 on **(A)** the TEER of HCAECs after exposure for 24 h (N = 2, n = 2–4), **(B)** monocyte adhesion to HCAECs 2 h after the addition of monocytes (N = 2, n = 2–4), and **(C)** monocyte migration through HCAECs 48 h after the addition of monocytes (N = 2, n = 2–4). Data represent the mean ±95%CI, and open circles in each group represent individual chips. Statistical analysis was performed using the Kruskal–Wallis test followed by Dunn’s test for all pairs of samples for joint rank testing with Bonferroni correction. Asterisks indicate a statistically significant difference, and their colors indicate which groups were compared pairwise (*p < 0.05, **p < 0.01). VoC, Vascular-on-a-Chip; IL, interleukin; TNF, tumor necrosis factor; MCP, monocyte chemotactic protein; TEER, trans-endothelial electrical resistance; HCAEC, human coronary artery endothelial cell; CI, confidence interval.

**FIGURE 2:**
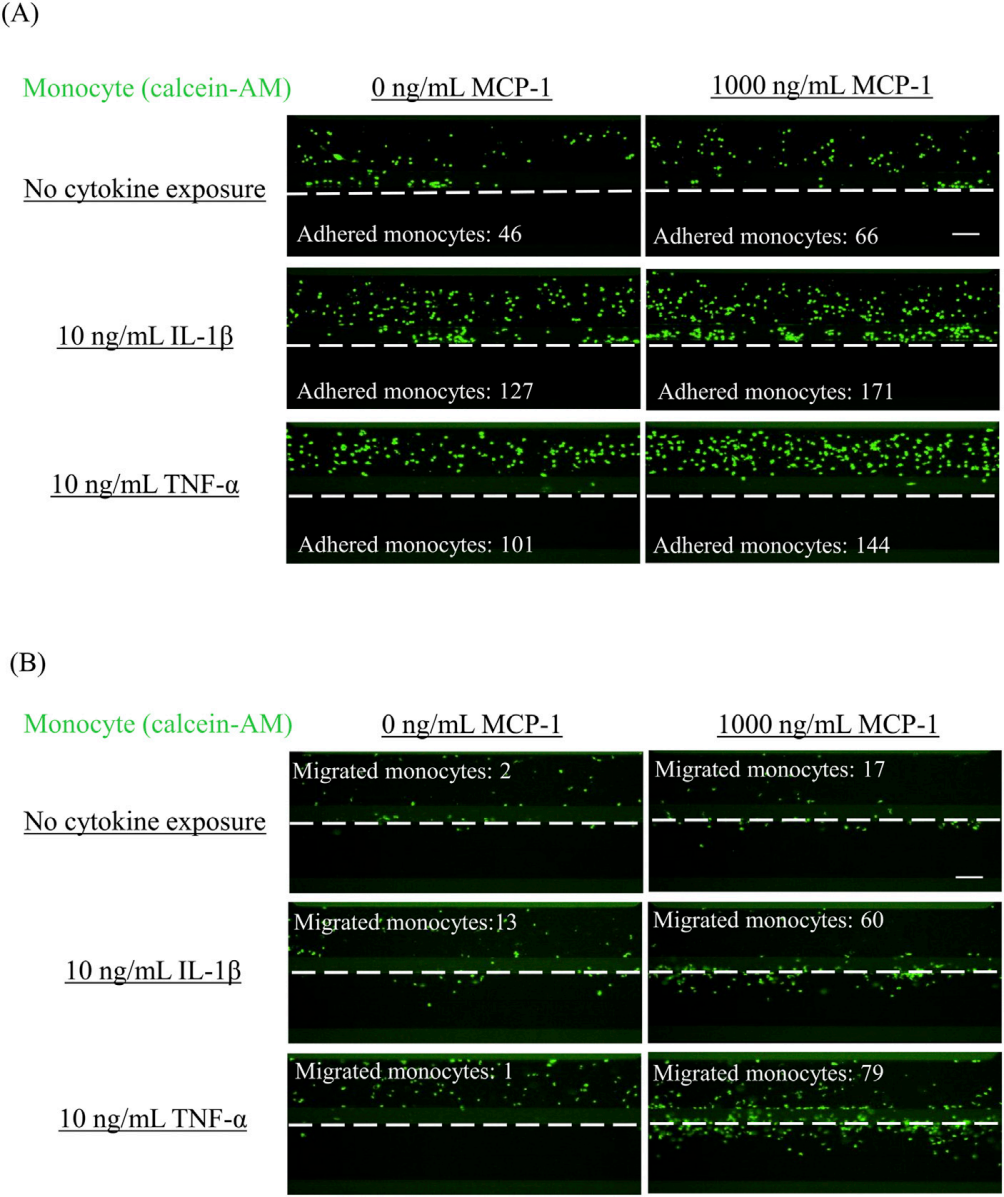
Representative images of adhered monocytes and migrated monocytes in VoC treated with proinflammatory cytokines Monocyte adhesion and migration were analyzed by fluorescent imaging. After allowing fluorescently labeled monocytes to flow for 2 h (monocyte adhesion) or 48 h (monocyte migration), images of **(A)** monocyte adhesion and **(B)** monocyte migration were obtained. Scale bar = 100 μm. White dashed line indicates the boundary between the tubule structure of HCAECs and the ECM region. VoC, Vascular-on-a-Chip; HCAEC, human coronary artery endothelial cell; ECM, extracellular matrix.

To confirm monocyte migration under inflammatory conditions, HCAECs were exposed to IL-1β and TNF-α at 10 ng/mL as positive controls. The TEER values of HCAECs exposed to IL-1β were significantly decreased compared with the non-treated group in the presence (*p* = 0.0050, *r* = 0.7692) and absence (*p* = 0.0225, *r* = 0.7692) of MCP-1 (Figure 1A). Regarding monocyte adhesion to the surface of HCAECs, exposure to 10 ng/mL TNF-α with MCP-1 induced a significant increase in adhered monocytes compared with the non-cytokine-treated groups in the presence (*p* = 0.0043, *r* = 0.7692) and absence (*p* = 0.0014, *r* = 0.7692) of MCP-1 (Figure 1B). In the monocyte migration assay, monocyte migration was significantly increased in the group co-treated with TNF-α and MCP-1 compared with the non-treatment group (*p* = 0.0064, *r* = 0.7710) (Figure 1C). The addition of MCP-1 significantly increased monocyte migration in the IL-1β- (*p* = 0.0274, *r* = 0.7701) and TNF-α (*p* = 0.0028, *r* = 0.7701)-treated groups compared with TNF-α treatment alone, suggesting a synergistic effect of MCP-1 on cytokine-induced monocyte migration. Representative images of monocyte adhesion and migration are shown in Figures 2A,B, respectively.

### 3.2 Changes in proinflammatory cytokine secretion by M1MΦs in response to tobacco product exposure

**FIGURE 3:**
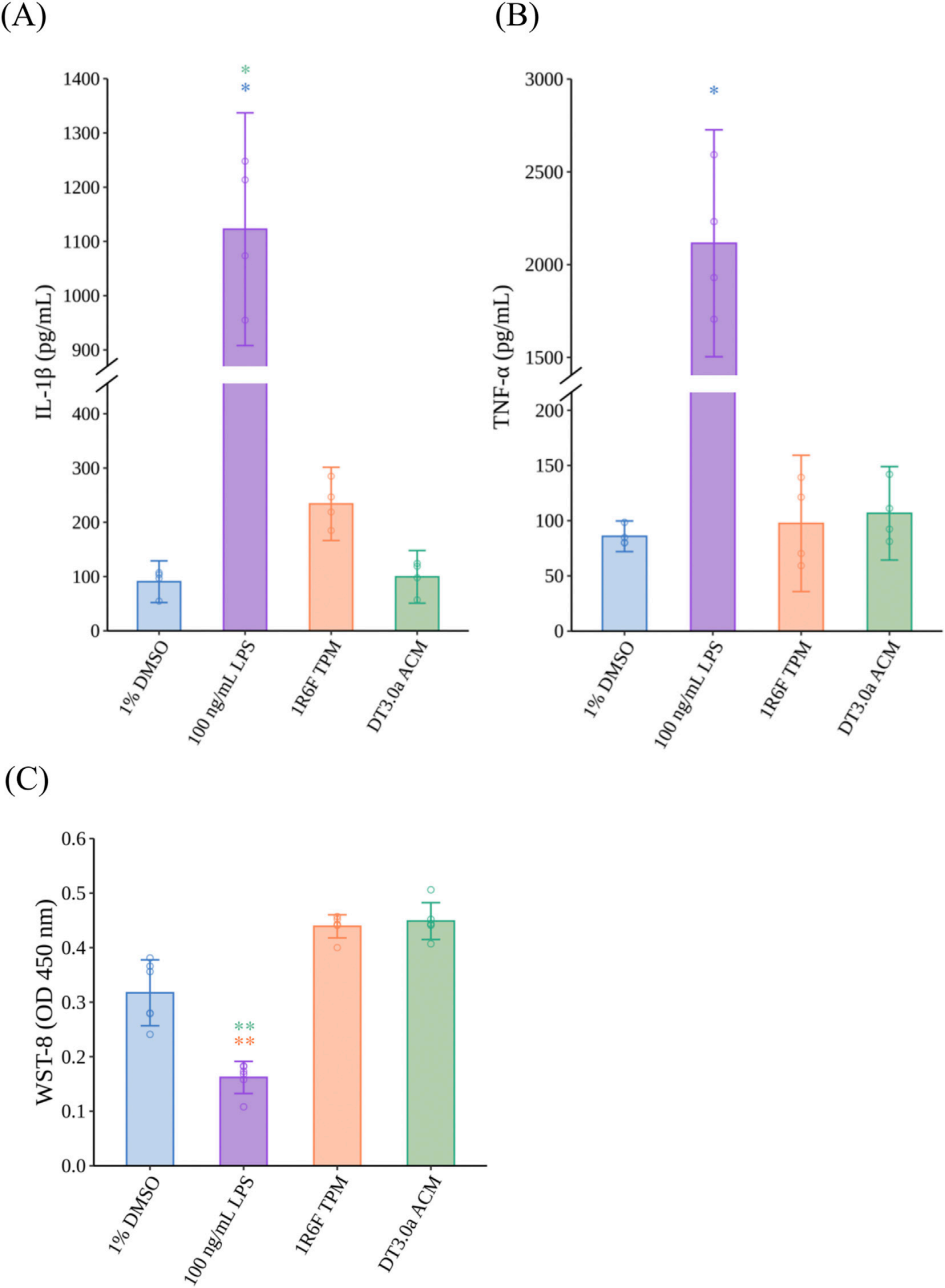
Proinflammatory cytokine secretion and cytotoxicity of M1 macrophages induced by LPS, 1R6F TPM, or DT3.0a ACM. **(A)** IL-1β and **(B)** TNF-α concentrations secreted from MΦ exposed to 100 ng/mL LPS, 400 μg/mL 1R6F TPM, or 400 μg/mL DT3.0a ACM (N = 2, n = 2). Data represent the mean ±95%CI and open circles represent the measured concentrations of each biological replicate. **(C)** Cytotoxicity assay of M1MΦs after exposure to test samples for 24 h (N = 2, n = 3). Data represent the mean ±95%CI, and open circles represent the absorbance value of each biological replicate. Statistical analysis was performed using the Kruskal–Wallis test followed by Dunn’s test for all pairs of samples for joint rank testing with Bonferroni correction. Asterisks indicate a statistically significant difference, and their colors indicate which groups were compared pairwise (*p < 0.05, **p < 0.01). LPS, lipopolysaccharide; TPM, total particulate matter; DT3.0a, Direct Heating Tobacco System Platform 3 Generation 0 version a; ACM, aerosol collected mass; IL, interleukin; TNF, tumor necrosis factor; MΦ, macrophage; DMSO, dimethyl sulfoxide; ELISA, enzyme-linked immunosorbent assay; CI, confidence interval.

The concentrations of IL-1β and TNF-α secreted from M1MΦs were measured to assess the immune response of M1MΦs exposed to 1R6F TPM, DT3.0a ACM, and LPS as a positive control (Figures 3A,B). Exposure to LPS increased the secretion of IL-1β (*p* = 0.0109, *r* = 0.7655) and TNF-α (*p* = 0.0451, *r* = 0.7655) compared with 1% DMSO (solvent control). Regarding tobacco products, although exposure to 1R6F TPM did not induce a significant difference compared with 1% DMSO (*p* = 0.3211), the concentration of IL-1β was approximately two-times higher than that of the 1% DMSO group and the effect size indicated a large change (*r* = 0.7655), whereas DT3.0a ACM induced a modest differential level of IL-1β secretion compared with that of 1% DMSO (*p* = 1.0000, *r =*0.2252). The concentrations of TNF-α released from M1MΦs exposed to 1R6F TPM (*p =*1.0000, *r* = 0.0000) or DT3.0a ACM (*p =*1.0000, *r* = 0.4593) were not significantly different from that induced by 1% DMSO. Cytotoxicity of M1MΦs was similar in the 1R6F TPM and DT3.0a ACM groups after 1 h of exposure (*p* = 1.0000, *r* = 0.0933). Although not statistically significant, the 1% DMSO group tended toward higher cytotoxicity relative to the 1R6F TPM (*p* = 0.2342, *r* = 0.8104) and DT3.0a ACM (*p* = 0.1399, *r* = 0.8449) groups, supported by large effect sizes. Although the comparison between the LPS-exposed M1MΦs and 1% DMSO-treated M1MΦs did not reach statistical significance (*p* = 0.9156), the potential cytotoxicity of LPS compared with 1% DMSO was indicated by the effect size (*r* = 0.8449).

### 3.3 The effect of LPS-exposed M1MΦ secretions on TEER and monocyte adhesion and migration

**FIGURE 4:**
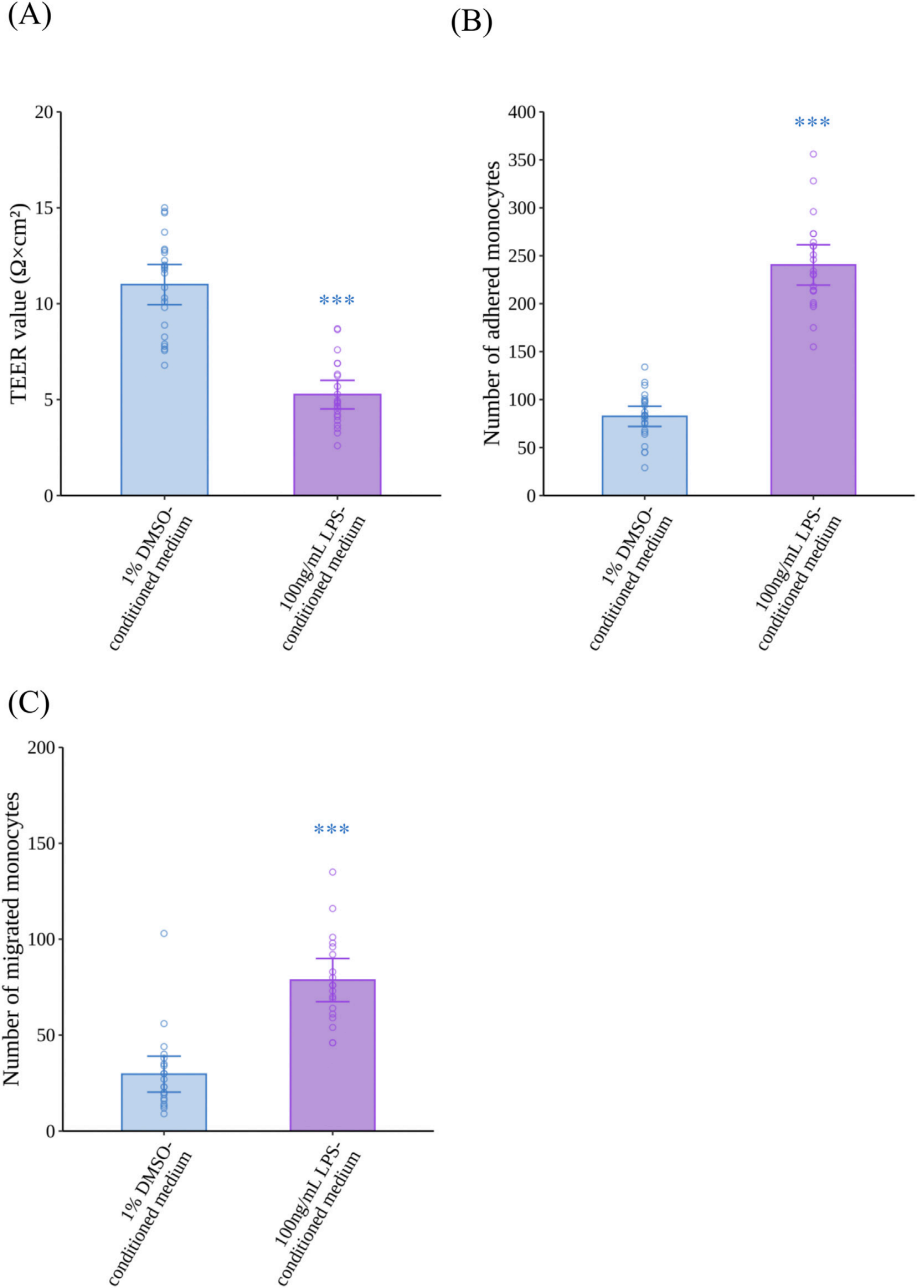
Effects of LPS-conditioned medium on the barrier integrity of HCAECs, and adhesion and migration of monocytes. **(A)** The TEER of HCAECs after 24 h of exposure to LPS-conditioned medium (N = 4, n = 5 or 6). **(B)** Monocyte adhesion to HCAECs 2 h after the addition of monocytes (N = 4, n = 5 or 6), and **(C)** monocyte migration through HCAECs 48 h after the addition of monocytes (N = 4, n = 5 or 6). Data represent the mean ±95%CI, and open circles in each group represent the value of each biological replicate. Statistical analysis was performed using the Mann–Whitney *U*-test. Asterisks indicate a statistically significant difference, and their colors indicate which groups were compared pairwise (***p < 0.001). LPS, lipopolysaccharide; HCAEC, human coronary artery endothelial cell; TEER, trans-endothelial electrical resistance; DMSO, dimethyl sulfoxide; CI, confidence interval.

To confirm our VoC model replicates monocyte migration induced by inflammatory activated M1MΦs, HCAECs were exposed to LPS-conditioned medium for 24 h to mimic inflammatory conditions in the vasculature of patients with atherosclerosis. LPS-conditioned medium significantly decreased the TEER of HCAECs compared with 1% DMSO-conditioned medium (*p* < 0.0001, *r* = 0.8025) (Figure 4A). Regarding monocyte adhesion and migration (Figures 4B,C, respectively), LPS-conditioned medium induced a significant increase in the number of adhered monocytes (*p* < 0.0001, *r* = 0.8545) and migrated monocytes (*p* < 0.0001, *r* = 0.7150) compared with 1% DMSO-conditioned medium. Representative fluorescence images of the monocyte adhesion and migration assays are shown in Supplementary Figures S6A, S6B, respectively.

### 3.4 Differential effects of CS and HTP aerosol via M1MΦs on endothelial dysfunction and monocyte migration

**FIGURE 5:**
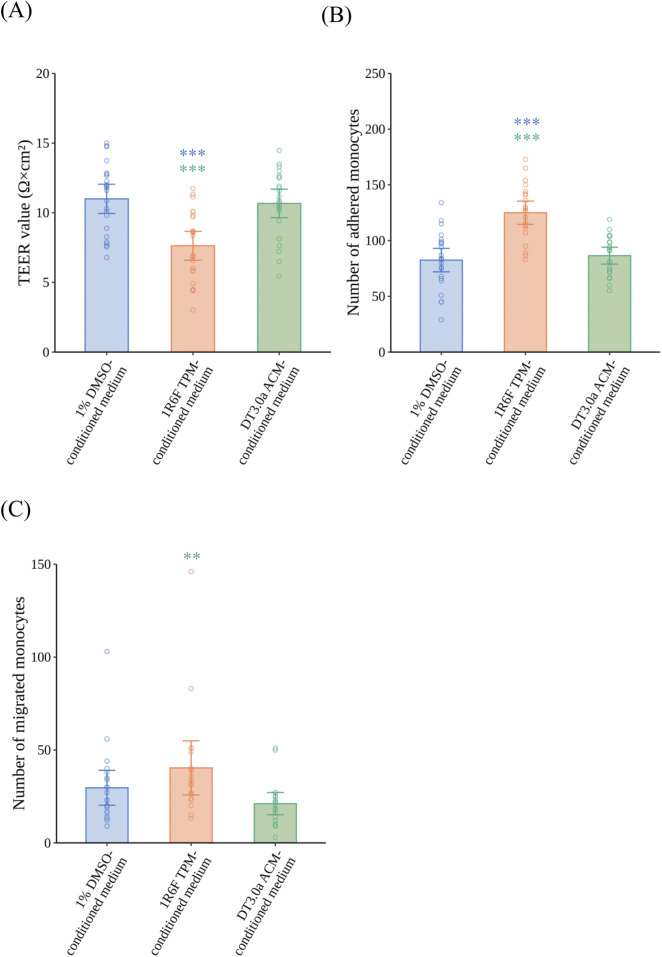
Effects of 1R6F TPM- and DT3.0a ACM-conditioned medium on the barrier integrity of HCAECs, and adhesion and migration of monocytes. VoC were exposed to 1R6F TPM-conditioned medium or DT3.0a ACM-conditioned medium for 24 h. **(A)** The TEER of HCAECs after 24 h of exposure to 1R6F TPM-conditioned medium or DT3.0a ACM-conditioned medium (N = 4, n = 5 or 6). After 24 h of exposure and the measurement of TEER, fluorescently labeled monocytes were added. **(B)** The number of monocytes adhered to HCAECs at 2 h (N = 4, n = 5 or 6) and **(C)** the number of monocytes migrated through HCAECs 48 h after the perfusion of monocytes (N = 4, n = 5 or 6). Data represent the mean ±95%CI, and open circles in each group represent the value of each biological replicate. Statistical analysis was performed using the Kruskal–Wallis test followed by Dunn’s test for all pairs of samples for joint rank testing with Bonferroni correction. Asterisks indicate a statistically significant difference, and their colors indicate which groups were compared pairwise (**p < 0.01, ***p < 0.001). TPM, total particulate matter; ACM, aerosol collected mass; DT3.0a, Direct Heating Tobacco System Platform 3 Generation 0 version a; HCAEC, human coronary artery endothelial cell; TEER, trans-endothelial electrical resistance; DMSO, dimethyl sulfoxide; CI, confidence interval.

After the VoC was exposed to 1R6F TPM- or DT3.0a ACM-conditioned medium for 24 h, TEER measurements (Figure 5A), and monocyte adhesion (Figure 5B) and monocyte migration assays (Figure 5C) were performed. 1R6F TPM-conditioned medium significantly decreased TEER compared with 1% DMSO-conditioned medium (*p* = 0.0002, *r* = 0.5640). In contrast, HCAECs exposed to DT3.0a ACM-conditioned medium maintained a similar level of TEER as that of the 1% DMSO-conditioned medium group after exposure (*p* = 1.0000, *r* = 0.0636). In the monocyte adhesion assay, 1R6F TPM-conditioned medium significantly increased the number of adhered monocytes compared with 1% DMSO-conditioned medium (*p* < 0.0001, *r* = 0.6723), whereas no significant increase in monocyte adhesion was induced by DT 3.0a ACM-conditioned medium compared with 1% DMSO-conditioned medium (*p* = 1.0000, *r* = 0.0652). In the monocyte migration assay, 1R6F TPM-conditioned medium increased the number of migrated monocytes compared with DT3.0a ACM-conditioned medium (*p* = 0.0054, *r* = 0.5026). There was a trend towards increased numbers of migrated monocytes in 1R6F TPM-conditioned medium compared with 1% DMSO-conditioned medium, indicated by a small effect size, but the difference was not statistically significant (*p* = 0.2979, *r* = 0.2593). DT3.0a ACM-conditioned medium and 1% DMSO-conditioned medium showed no statistically significant difference in monocyte migration, although a small effect was indicated (*p* = 0.3711, *r* = 0.2422). Representative fluorescence images of the monocyte adhesion and migration assays are shown in Supplementary Figures S7A, S7B, respectively.

## 4 Discussion

Previously, we established a method to assess monocyte adhesion induced by inflammation under microfluidic conditions (Ohashi et al., 2023). We used this method to evaluate the reduced risk potential of an HTP in relation to the development of atherosclerosis. According to the AOP approach, elucidating the sequence of key molecular and cellular events provides a more precise understanding of the causality of toxic effects. Therefore, studying the later stages of atherosclerosis will further our understanding of the reduced risk potential of HTPs for the development of atherosclerosis in individual users. Monocyte migration is influenced by monocyte adhesion and impaired endothelial barrier integrity, and migrated monocytes play a crucial role in plaque formation (Ley et al., 2011; Pamukcu et al., 2010). Therefore, we revised our assessment model to assess endothelial barrier integrity and monocyte migration with OrganoPlate^®^.

Previous methods to evaluate cell migration used an insert culture system with a membrane of arbitrary pore size under static conditions (Huwait et al., 2022; Dorenkamp et al., 2023; Zhou et al., 2018). However, these studies indicated that shear stress caused by blood flow had a critical effect on the behavior of monocytes and molecular regulation. Chiu et al. reported that shear stress altered the expressions of adhesion molecules that contributed to the attachment of monocytes to VECs prior to monocyte migration (Chiu et al., 2004). We previously demonstrated monocyte adhesion at more clinically relevant cytokine concentrations using a VoC model with M0MФs that replicated the shear stress in regions prone to atherosclerosis compared with other studies using static conditions (Ohashi et al., 2023). Here, we demonstrated that IL-1β and TNF-α impaired the barrier functions of VECs and monocyte adhesion (Figures 1A,B), whereas cytokines alone did not sufficiently induce monocyte migration in the IL-1β (*p* = 1.000, *r* = 0.3442) and TNF-α groups (*p* = 1.0000, *r* = 0.3063) (Figure 1C). Monocyte migration was induced by adding MCP-1 to the bottom lane, and proinflammatory cytokines and MCP-1 acted synergistically on the progression of monocyte adhesion to migration. These results are consistent with the general understanding of the early stages of atherogenesis; thus, our model appropriately reproduced the sequence of early atherosclerosis development. In addition, we found that IL-1β, even at 100 pg/mL in the presence of MCP-1, elicited pronounced effects on the early events in atherosclerosis (Supplementary Figure S3). Taken together, adding MCP-1 to the bottom lane did not reduce the sensitivity of endothelial dysfunction and monocyte adhesion as expected, which made it possible to use VoC to evaluate monocyte migration (Ohashi et al., 2023).

When considering the mechanisms by which tobacco product use is associated with the progression of atherosclerosis, it should be noted that tobacco smoke affects the vascular system through various mediators. During inflammatory responses, MΦs in the respiratory tract play a crucial role by secreting proinflammatory cytokines induced by smoking (Lugg et al., 2022; Churg et al., 2002) and these cytokines enter the bloodstream and induce endothelial dysfunction in vascular regions (Tamagawa et al., 2008). Our previous report assessed the effectiveness of M0MΦs and M1MΦs to stimulate atherosclerotic reactions and found that incorporating M1MΦs into the VoC system represented the inflammatory conditions observed during atherogenesis, which can enhance the *in vitro* recapitulation related to inhalable substances by using RNA sequencing (Ohashi et al., 2024). Therefore, we used M1MΦs for the following assessments. Compared with a previous study using M0MΦs (Ohashi et al., 2023), M1MΦs secreted similar levels of TNF-α after stimulation with 100 ng/mL LPS (*cf.* 1,000 ng/mL in a previous study) (Figure 3B). Moreover, IL-1β secretion was twice as high after LPS treatment (Figure 3A). The extent of differentiation to M1MΦs was not examined in this study, but these data suggest that adding M1MΦs could make the model more sensitive to proinflammatory challenge. This modification was effective in assays related to the early stages of atherosclerosis (Figure 4), indicating IL-1β had more pronounced effects on the monocyte adhesion and migration assays than TNF-α, especially at low concentrations (Supplementary Figures S3B, 3C). In addition, 1% DMSO-conditioned medium impaired the barrier integrity of VECs compared with non-treatment controls (Supplementary Figure S5A). This may be explained by M1MΦs exhibiting a proinflammatory phenotype and constitutively secreting inflammatory cytokines, even in the absence of strong stimulation (Arango and Descoteaux, 2014), which likely affected the permeability of endothelial cells. The advantage of this exposure method is that the model can sensitively capture the immune cell-mediated effects of test substances under inflammatory conditions, which elicit further proinflammatory responses leading to the progression of atherogenesis.

Based on our previous report, exposing VECs to HTP aerosol via M1MΦs did not activate atherosclerotic signaling pathways compared with CS at the transcriptomic level (Ohashi et al., 2024). However, to understand the reduced risk potential of HTPs in the context of atherosclerosis, it is essential to expose established *in vitro* models to CS- or HTP aerosol-derived samples and verify whether they change the key events related to atherosclerosis susceptibility at the tissue level. In this study, we exposed VoC to conditioned media with M1MΦs based on our previous studies that demonstrated this exposure method reflected tissue-resident MΦ-mediated vascular responses to tobacco products (Ohashi et al., 2023). Moreover, we used M1MΦs instead of M0MΦs as used in our previous *in vitro* assessment (Ohashi et al., 2024). This allowed us to observe the reduced risk potential of HTPs under chronic inflammatory conditions, which are considered more relevant to atherosclerosis (Ajoolabady et al., 2024). 1R6F TPM and DT3.0a ACM were extracted using DMSO, so the samples exposed to M1MΦs contained DMSO, which is cytotoxic at certain concentrations (Hassan and Ahmad, 2024). Therefore, changes in cell viability were observed in M1MΦs exposed to 1% DMSO as a solvent control compared with the non-treatment control (*p* = 0.0082, *r* = 0.3896) (Supplementary Figure S4C). To normalize this effect, the concentration of DMSO was standardized to 1% in all samples exposed to M1MΦs, including TPM and ACM. Our data showed that exposing DT3.0a ACM to M1MΦs reduced the production of a representative proinflammatory cytokine, IL-1β, compared with 1R6F TPM (*p* = 0.3211, *r* = 0.7655), although it was a limited practical difference to the level induced by the 1% DMSO solvent control (*p* = 1.0000, *r =*0.2252) (Figures 3A,B). These data suggest that the biological impact of HTP aerosol on M1MΦ-mediated inflammatory responses was negligible. Several studies reported that the HTPs we used in this study produced an aerosol containing fewer HPHCs than CS because it heats tobacco sticks rather than combusting tobacco to produce an aerosol (Forster et al., 2018; Hashizume et al., 2023; Maeder and Jeannet, 2025). HPHCs stimulate immune cells when they enter the body, triggering inflammatory responses. Although differences in heating methods for each HTP must be considered, our data suggest our HTP had a low impact on cytokine release from tissue-resident MΦs induced by inflammatory responses because of the reduction in HPHCs.

To compare the effects of CS or HTP aerosol on monocyte migration induced by inflammatory responses, we exposed the VoC to conditioned media. In the current study, DT3.0a ACM-conditioned medium had a lower biological impact on the TEER values of VECs (*p* = 0.0002, *r* = 0.5640) and monocyte adhesion (*p* < 0.0001, *r* = 0.6723) than 1R6F TPM-conditioned medium, which was similar to the 1% DMSO-conditioned medium group regarding TEER values (*p* = 1.0000, *r* = 0.0636) and monocyte adhesion (*p* = 1.0000, *r* = 0.0652) (Figures 5A,B). Monocyte migration caused by exposure to DT3.0a ACM-conditioned medium was also significantly lower than that caused by 1R6F TPM-conditioned medium (*p* = 0.0054, *r* = 0.5026) (Figure 5C). These results are in line with the concentration of IL-1β being lower in DT3.0a ACM-conditioned medium than 1R6F TPM-conditioned medium. Therefore, it is plausible that the causal sequence from the elicitation of inflammation to monocyte migration in the development of atherosclerosis is reduced for DT3.0a compared with combustible cigarettes. These results confirm that HTP has a reduced risk potential for atherosclerosis related to its diminished capacity to trigger monocyte migration via inflammatory responses mediated by immune cells.

When interpreting the data obtained in this study, it is important to note the substantial discrepancy between the experimental exposure conditions and real-world tobacco smoke exposure scenarios. In the current study, we exposed M1MΦs to 400 μg/mL 1R6F TPM or DT3.0a ACM. According to one clinical study, the highest concentration of nicotine in plasma was 21.0–39.2 ng/mL in CC smokers and 16.1–28.7 ng/mL in HTP smokers (Nishihara et al., 2024). If all TPM or ACM, including nicotine, is absorbed by the alveoli, based on a previous study (Hashizume et al., 2023), the TPM or ACM concentration in the alveolar region will be 494.2–922.5 ng/mL in CC smokers and 514.5–917.1 ng/mL in HTP smokers. Although a direct comparison is not fully appropriate because of differences in sample collection and extraction with DMSO, it should be noted that the exposure concentration used in our study was more than 1,000 times higher than the estimated levels in human tissues. Additionally, when focusing on the correlation between an unhealthy lifestyle, including smoking and the risk of CVD, it should be noted that the development of atherosclerosis associated with repeated exposure to risk factors occurs over decades (Ding et al., 2019; Whisnant et al., 1990). In summary, the high concentration and short-term exposure method conducted in this study was intended as an accelerated test, which may be useful for screening the potential biological impact of tobacco products on atherosclerosis. However, it should be clearly noted that such findings cannot be directly extrapolated to human disease risk assessment. Therefore, *in vitro* repeated and long-term exposure incorporating these risk factors at lower concentrations to fully recapitulate their chronic impact on atherosclerosis may help elucidate a more realistic reduced-risk potential of HTPs. In this study, the entire process from cell seeding to evaluation was completed within 1 week, and the VoC model was not cultured beyond this period. However, gaining a proper understanding of the lifespan of the VoC model and developing a method capable of long-term culture may be valuable for future research by bridging the gap between *in vitro* experiments and the human condition.

## 5 Conclusion

We established an evaluation method for monocyte migration induced by inflammation using a vascular OoC platform. Our model recapitulates the impaired barrier integrity, and monocyte adhesion and migration induced by inflammation under microfluidic conditions with indirect exposure to test substances via M1MΦs to demonstrate the interaction between immune cells and VECs. Using this model to assess tobacco products, it was found that HTP aerosol induced lower inflammatory responses from M1MΦs compared with CS, leading to reduced impairment of barrier integrity, and reduced monocyte adhesion and migration. Additionally, the biological response to HTP aerosol was comparable with that of the solvent control, suggesting the reduced risk potential of HTP for atherosclerosis.

## Data Availability

The raw data supporting the conclusions of this article will be made available by the author, without undue reservation.

## Supplementary material

Supplementary Figure S1 A schematic overview of OrganoPlate^®^ 3-lane 40. **(A)** An overview of a chip on the OrganoPlate^®^ 3-lane 40. A plate contains 40 chips based on the platform of a 384-well plate. Each lane has inlets and outlets to allow the addition of cells or reagents. **(B)** A schematic view of the tubules of HCAECs from the top and from the side. HCAECs, human coronary artery endothelial cells; ECM, extracellular matrix.

Supplementary Figure S2 Barrier formation of HCAECs cultured in OrganoPlate^®^ 3-lane 40. **(A)** The TEER of HCAECs seeded in OrganoPlate^®^ on day 5 cultured with MV2 or OrganoMedium (N = 3, n = 6 or 7). Cell-free chips were below the measurement limit of the OrganoTEER^®^ (N = 2, n = 3). Open circles in each group represent the value of each biological replicate. Bars represent the mean, and error bars indicate 95%CI. Statistical analysis was performed using the Mann–Whitney *U*-test. Asterisks indicate a statistically significant difference (****p* < 0.001). **(B)** Representative fluorescence image of VoC on day 5 at the best focused position stained with ZO-1 (orange) and nuclei stained with Hoechst 33342 (blue). TEER, trans-endothelial electrical resistance; HCAEC, human coronary artery endothelial cell; VoC, Vascular-on-a-Chip; ZO-1, Zonula Occludens-1; n.d., not detected; CI, confidence interval.

Supplementary Figure S3 Dose-response of the TEER, and monocyte adhesion and migration to proinflammatory cytokines under the conditions of MCP-1 addition. **(A)** The TEER of HCAECs after 24 h of exposure to proinflammatory cytokines (N = 2, n = 2 or 3). **(B)** The number of adhered monocytes on HCAECs 2 h after the addition of monocytes (N = 2, n = 2 or 3). **(C)** The number of monocytes that migrated through HCAECs with the addition of MCP-1 48 h after the addition of monocytes (N = 2, n = 2 or 3). Data represent the mean ±95%CI. Statistical analysis was performed using the Kruskal–Wallis test followed by Dunn’s test with a control for joint rank tests (control: 0 ng/mL of IL-1β or TNF-α) with Bonferroni correction. Asterisks indicate a statistically significant difference compared with respective solvent controls (**p* < 0.05, ***p* < 0.01, ****p* < 0.001). TEER, trans-endothelial electrical resistance; HCAEC, human coronary artery endothelial cell; MCP, monocyte chemotactic protein; IL, interleukin; TNF, tumor necrosis factor; CI, confidence interval.

Supplementary Figure S4 Cytotoxicity of unexposed M1 macrophages. **(A)** IL-1β and **(B)** TNF-α concentrations secreted from M1MΦ exposed to 1% DMSO or non-treated M1MΦ (non-treatment control) (N = 2, n = 2). **(C)** Cytotoxicity assay of M1MΦs after exposure to 1% DMSO for 24 h or unexposed M1MΦs (non-treatment control) (N = 2, n = 3). Data represent the mean ±95%CI, and open circles represent the absorbance value of each biological replicate. Statistical analysis was performed using the Mann–Whitney *U*-test. Asterisks indicate a statistically significant difference (***p* < 0.01). IL, interleukin; TNF, tumor necrosis factor; MΦs, macrophages; DMSO, dimethyl sulfoxide; CI, confidence interval.

Supplementary Figure S5 The TEER, and monocyte adhesion and migration of non-treated VoCs. As a non-treatment control, VoCs were cultured with OrganoMedium for 24 h. **(A)** The TEER of HCAECs after 24 h of cultivation (N = 4, n = 5 or 6). **(B)** Monocyte adhesion to HCAECs 2 h after the addition of monocytes (N = 4, n = 5 or 6), and **(C)** monocyte migration through HCAECs 48 h after the addition of monocytes (N = 4, n = 5 or 6). Four separate experiments with four to six chips in each experiment were performed (n = 17–24). Data represent the mean ±95%CI, and open circles in each group represent the value of each biological replicate. Statistical analysis was performed using the Mann–Whitney *U*-test. Asterisks indicate a statistically significant difference (****p* < 0.001). VoC, Vascular-on-a-Chip; TEER, trans-endothelial electrical resistance; HCAEC, human coronary artery endothelial cell; DMSO, dimethyl sulfoxide; CI, confidence interval.

Supplementary Figure S6 Monocyte adhesion and monocyte migration induced by exposure to LPS-conditioned medium. Representative fluorescence images of **(A)** monocytes adhered to HCAECs 2 h after the addition of monocytes, and **(B)** monocytes migrated through HCAECs 48 h after the addition of monocytes. White dashed line indicates the boundary between the top and middle lane. Scale bar = 100 μm. HCAEC, human coronary artery endothelial cell; LPS, lipopolysaccharide; DMSO, dimethyl sulfoxide.

Supplementary Figure S7 Monocyte adhesion and migration induced by exposure to 1R6F TPM- or DT3.0a ACM-conditioned medium. Representative fluorescence images of **(A)** monocytes adhered to HCAECs 2 h after the addition of monocytes, and **(B)** monocytes migrated through HCAECs 48 h after the addition of monocytes. White dashed line indicates the boundary between the top and middle lane. Scale bar = 100 μm. TPM, total particulate matter; ACM, aerosol collected mass; DT3.0a: Direct Heating Tobacco System Platform 3 Generation 0 version a; HCAEC, human coronary artery endothelial cell; DMSO, dimethyl sulfoxide.
