# Supplementary material for: Monocyte migration assay using a vascular-on-a-chip model and its utilization for the evaluation of a heated tobacco product
Source: Front Toxicol. 2025 Dec 4;7:1658093. doi: 10.3389/ftox.2025.1658093 (PMC12711516; doi:10.3389/ftox.2025.1658093)
Supplement: Supplementary file 1 [file Presentation1.pptx]

## Slide 1
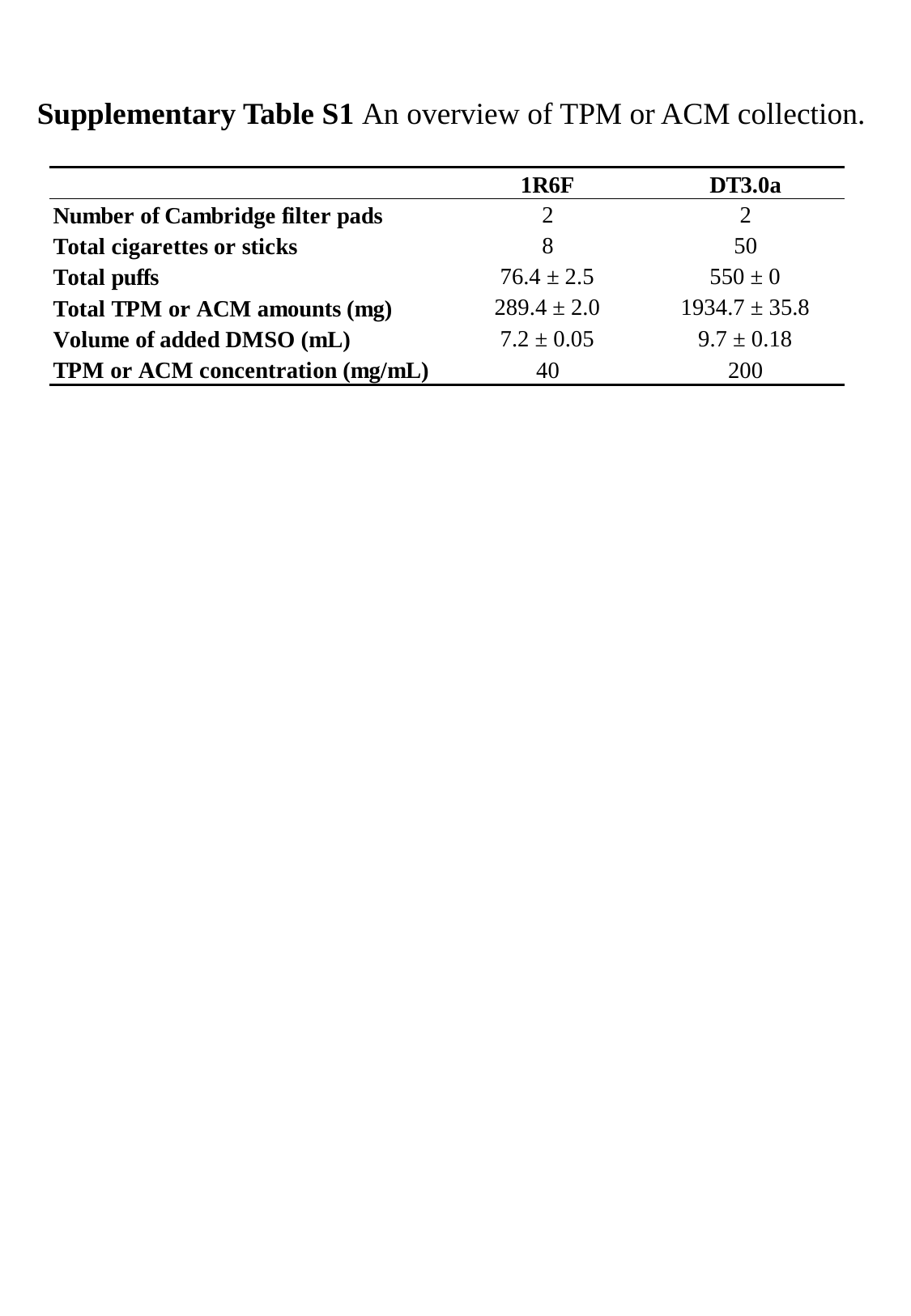

Supplementary Table S1 An overview of TPM or ACM collection.

## Slide 2
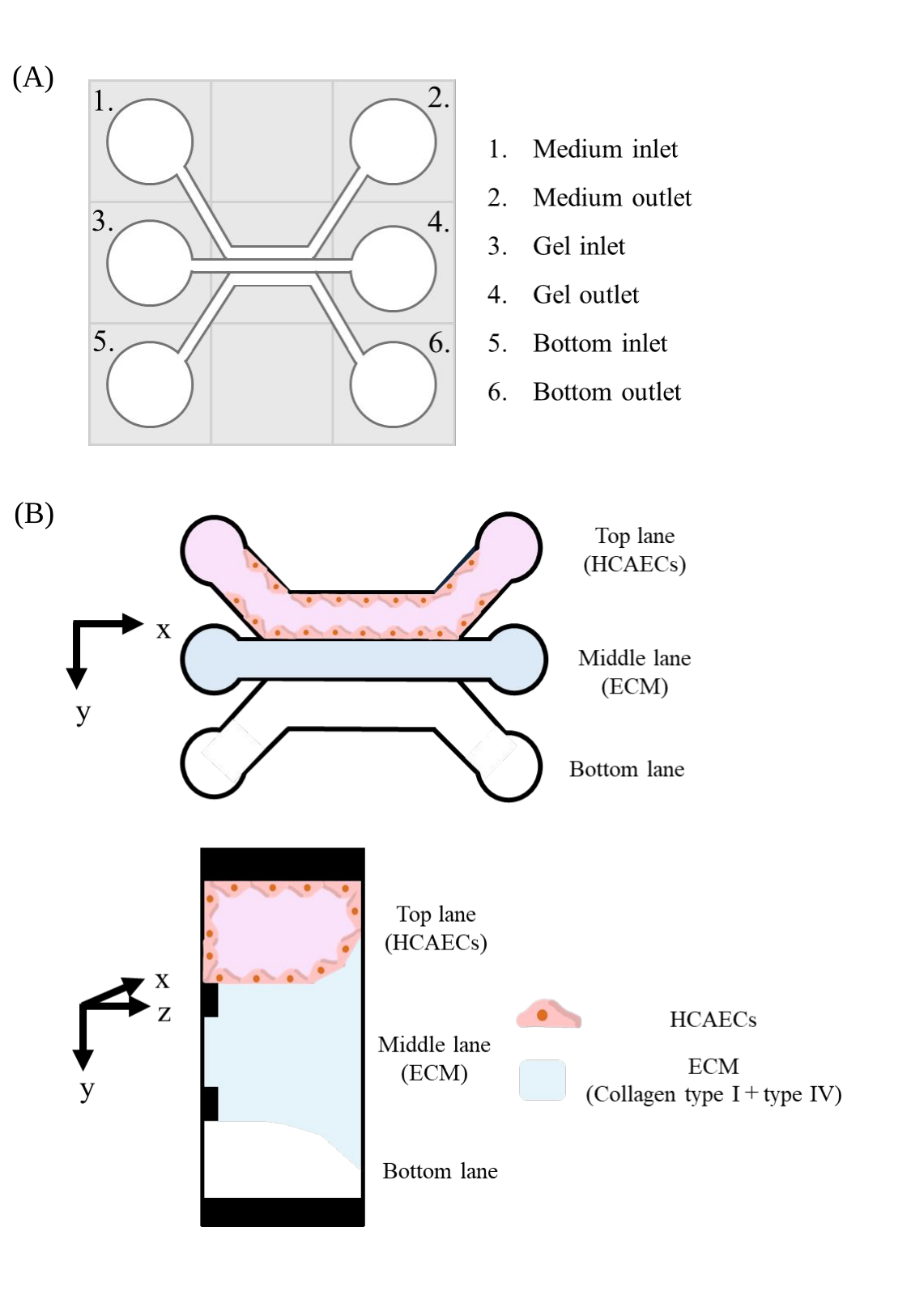

(A)
(B)

## Slide 3
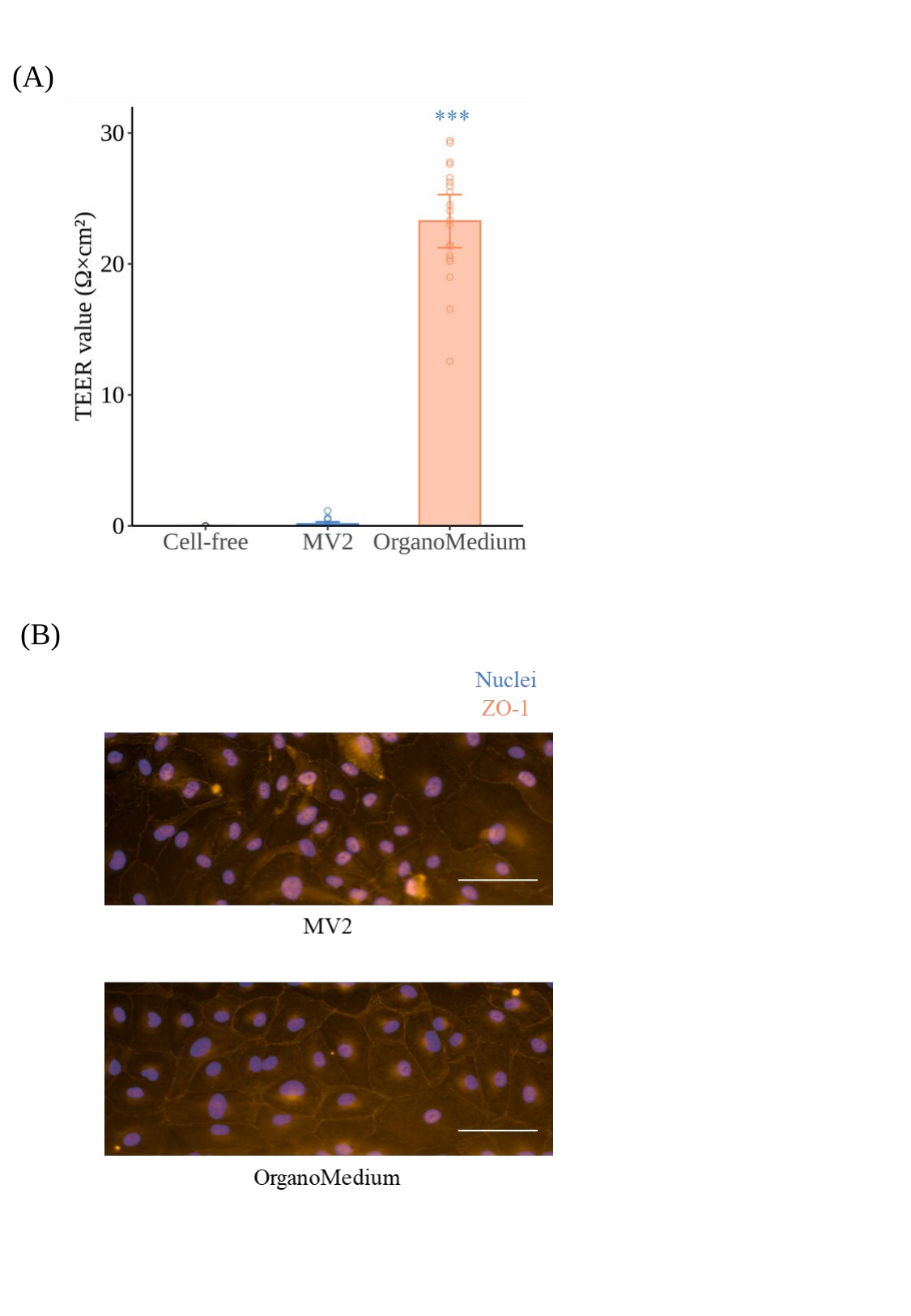

(A)
(B)

## Slide 4
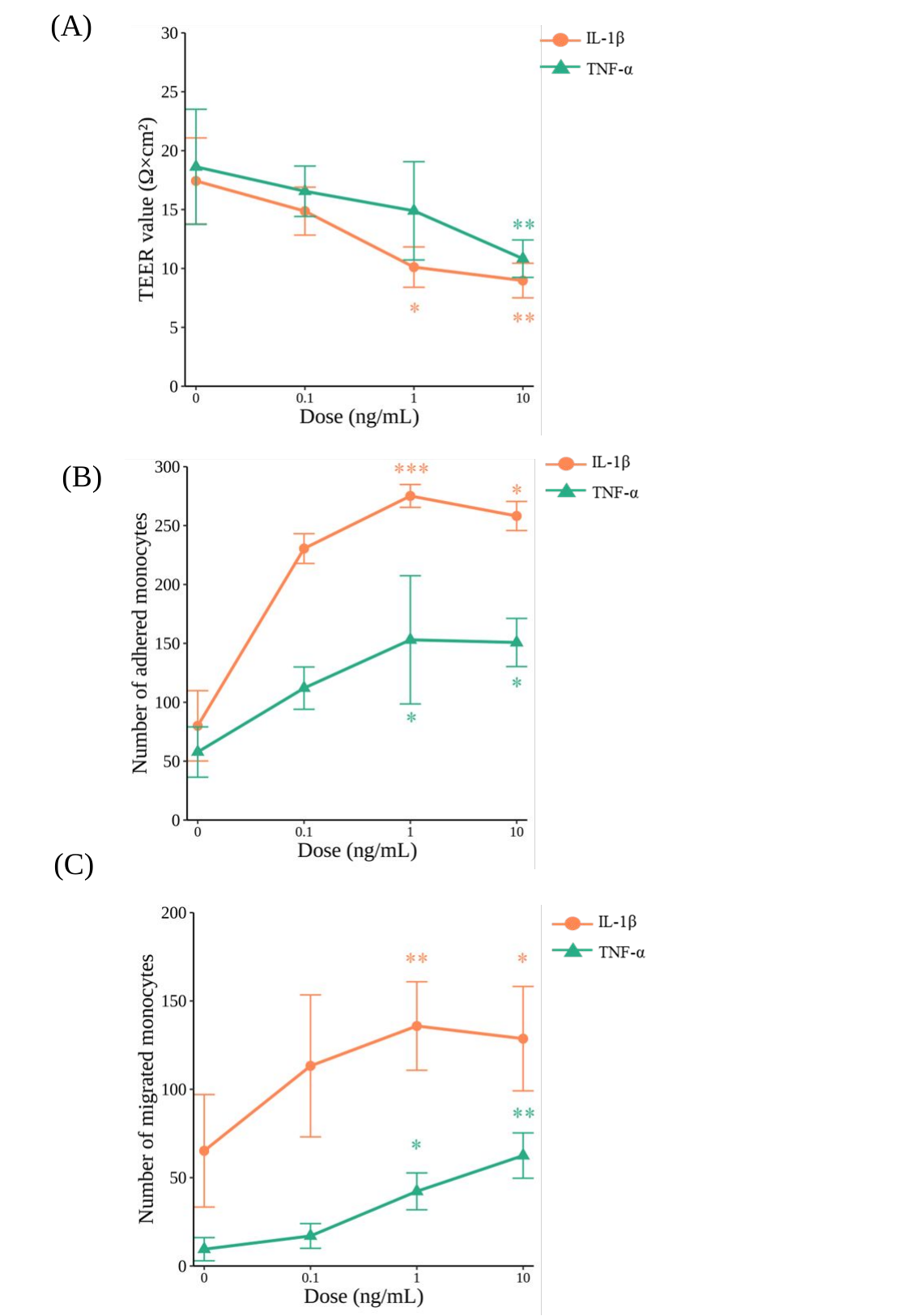

(A)
(B)
(C)

## Slide 5
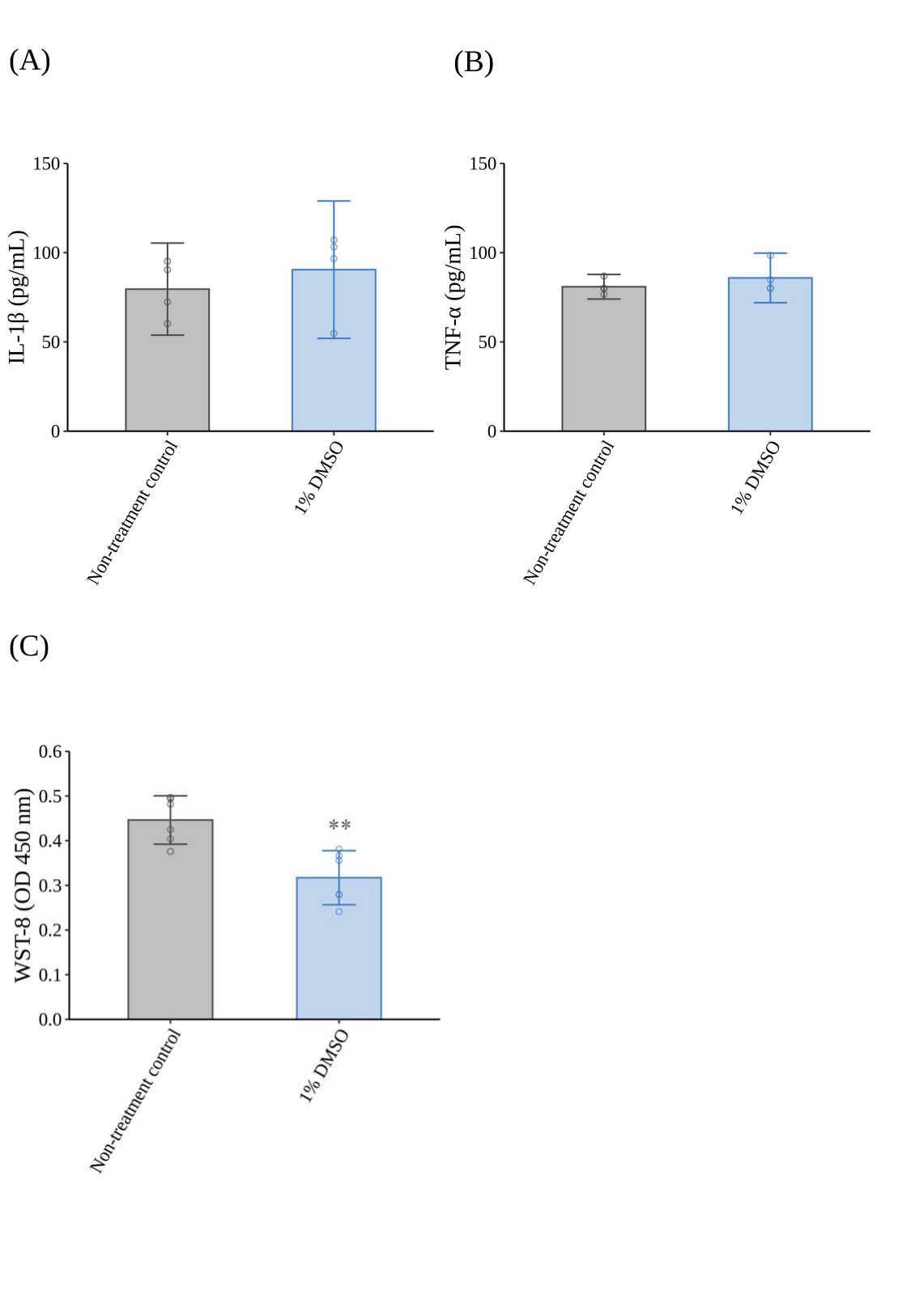

(A)
(B)
(C)

## Slide 6
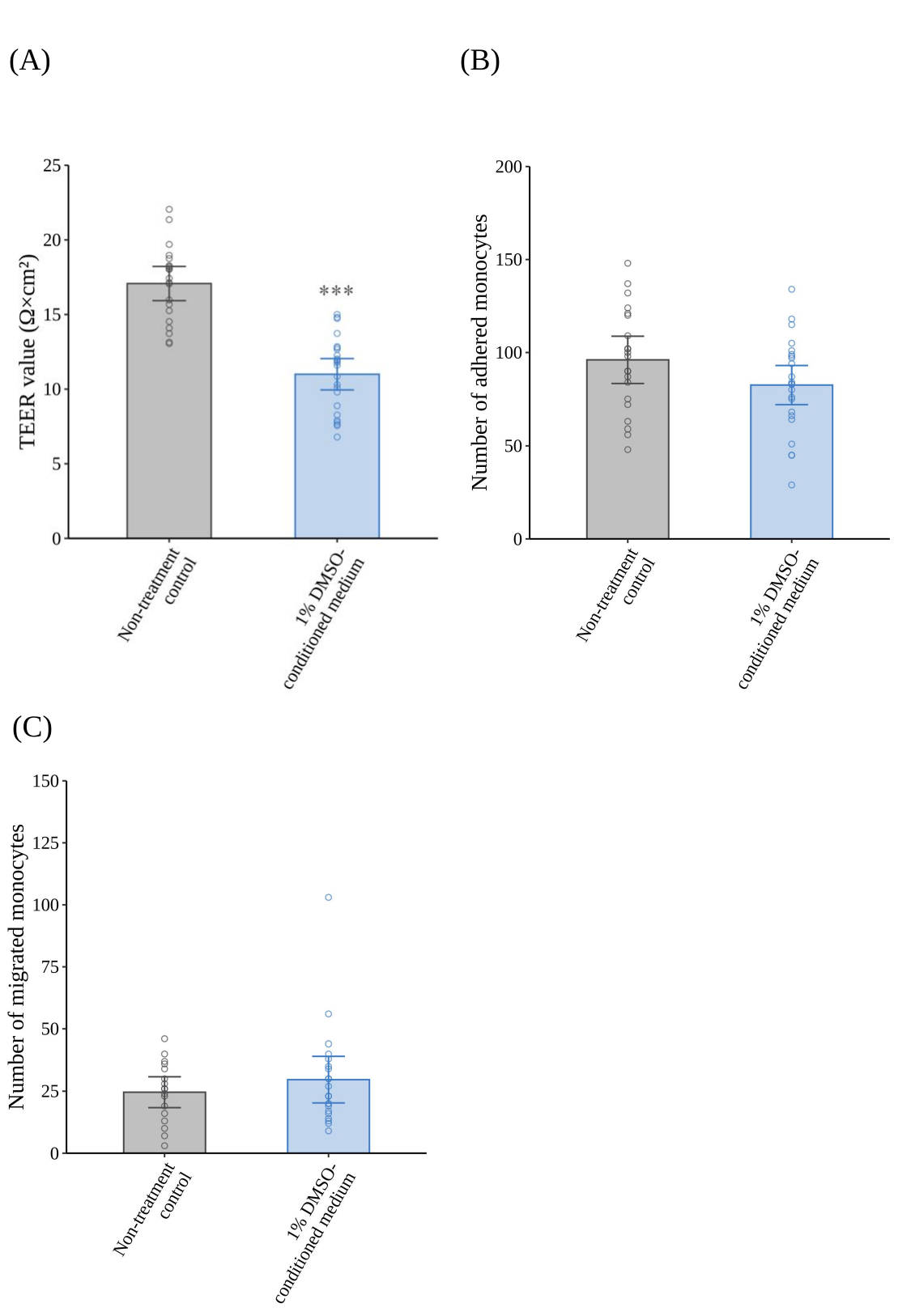

(A)
(B)
(C)

## Slide 7
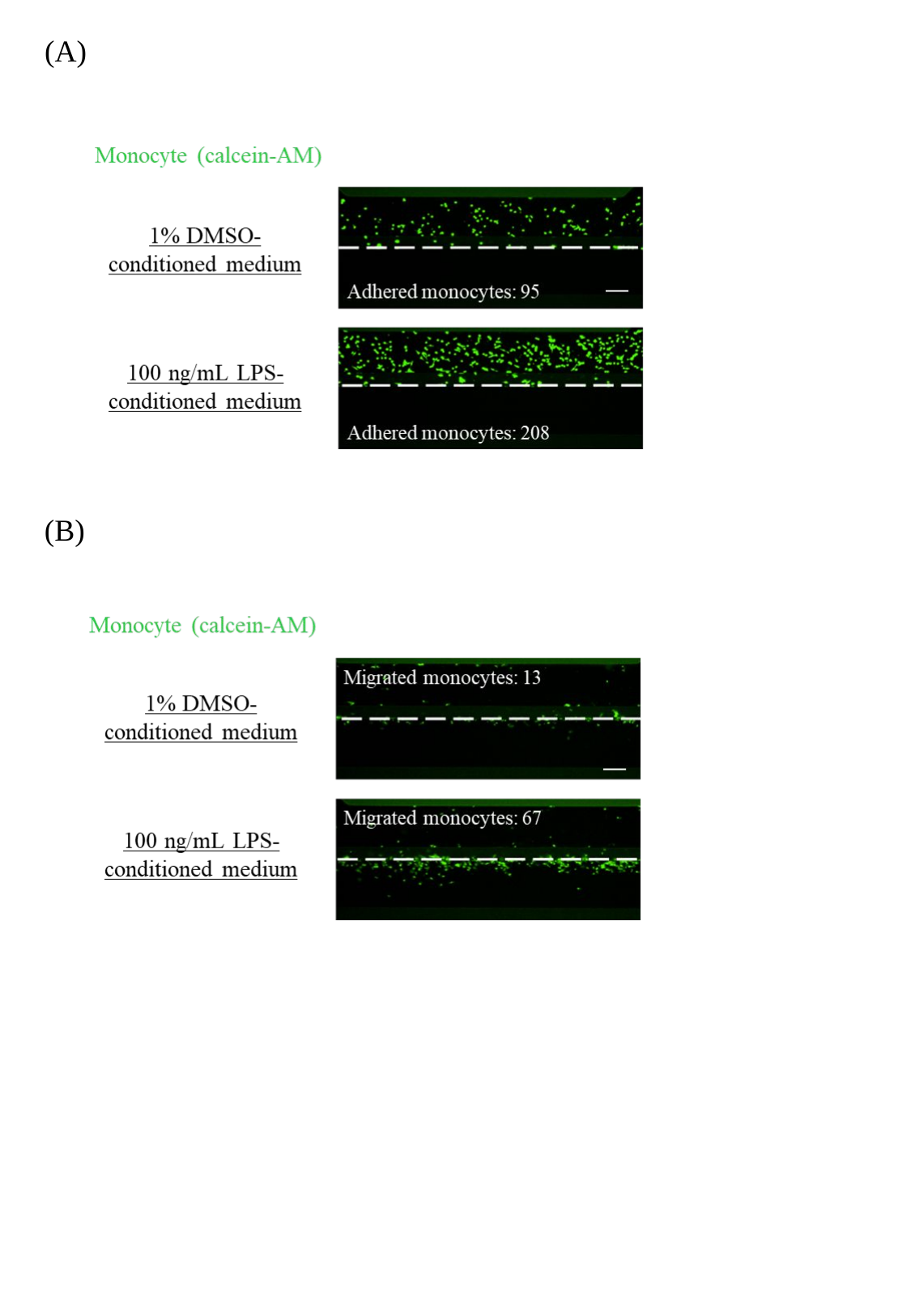

(A)
(B)

## Slide 8
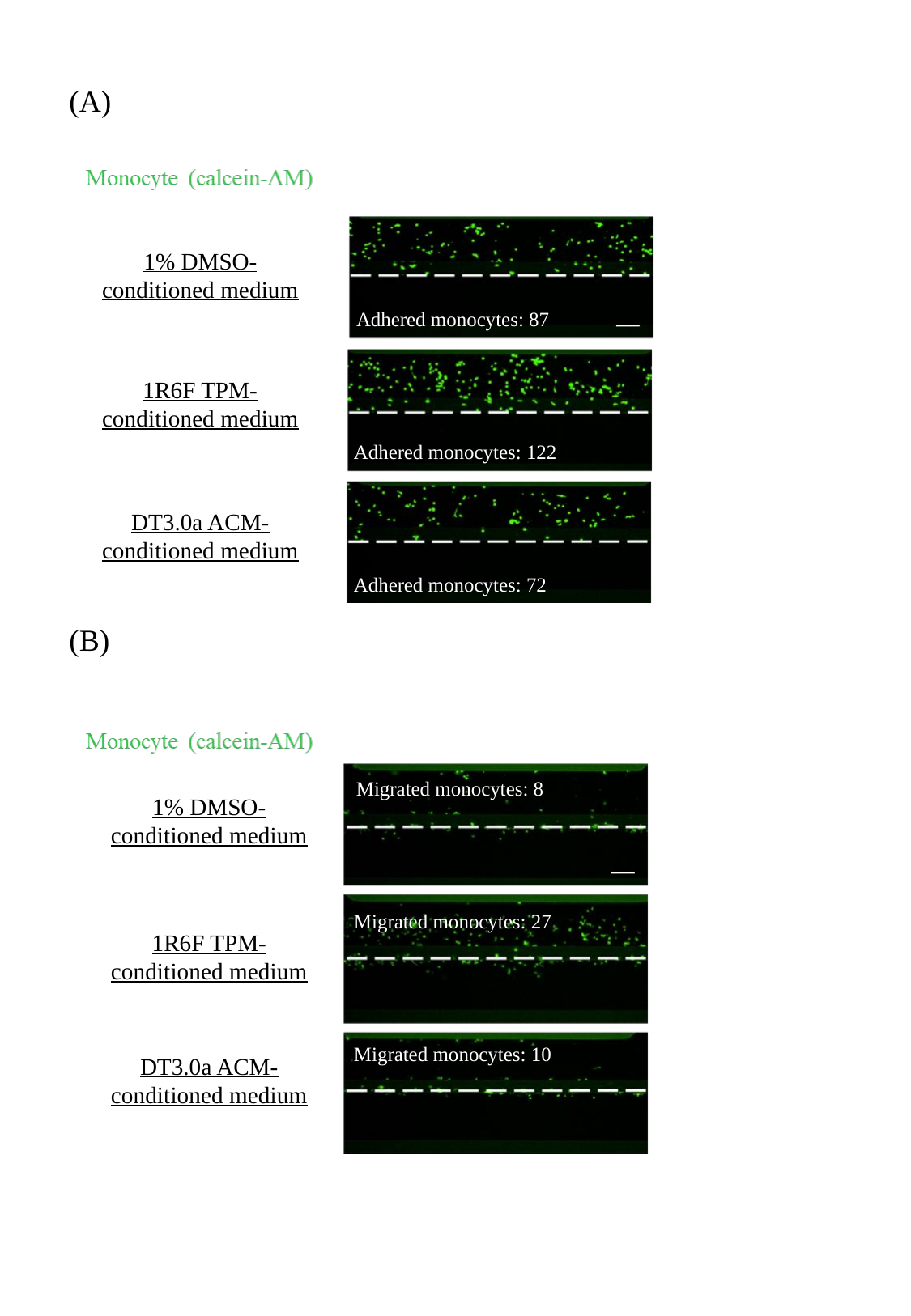

(A)
1% DMSO-
conditioned medium
Adhered monocytes: 87
1R6F TPM-
conditioned medium
Adhered monocytes: 122
DT3.0a ACM-
conditioned medium
Adhered monocytes: 72
(B)
Migrated monocytes: 8
1% DMSO-
conditioned medium
Migrated monocytes: 27
1R6F TPM-
conditioned medium
Migrated monocytes: 10
DT3.0a ACM-
conditioned medium
